# Prolonged molecular control during intermittent reduced‐intensity blinatumomab maintenance in a transplant‐ineligible patient with Philadelphia chromosome‐positive acute lymphoblastic leukaemia and *IKZF1* deletion

**DOI:** 10.1002/cti2.70121

**Published:** 2026-09-21

**Authors:** Xiaoqian Duan, Jinjun Yang, Yufei Yang, Shuwen Guo, Yu Wu

**Affiliations:** ^1^ Department of Hematology and Institute of Hematology West China Hospital, Sichuan University Chengdu China; ^2^ West China School of Medicine Sichuan University Chengdu China

**Keywords:** blinatumomab, *IKZF1* deletion, measurable residual disease, Philadelphia chromosome‐positive acute lymphoblastic leukaemia, T‐cell immunity

## Abstract

**Objectives:**

Long‐term disease control in transplant‐ineligible patients with high‐risk Philadelphia chromosome‐positive acute lymphoblastic leukaemia (Ph^+^ ALL) remains difficult, particularly when adverse genetic lesions and acquired kinase domain mutations coexist.

**Methods:**

Clinical, cytogenetic and molecular records were retrospectively reviewed. *BCR::ABL1* transcripts were monitored by quantitative PCR, and peripheral lymphocyte subsets were followed longitudinally. Public transcriptomic data (GSE196463) were analysed to compare T‐cell expression profiles under continuous versus intermittent bispecific antibody exposure.

**Results:**

A 64‐year‐old man with Ph^+^ ALL harbouring a heterozygous *IKZF1* deletion relapsed while receiving dasatinib, with emergence of an ABL1 E255K/V mutation. Although this P‐loop mutant retains sensitivity to dasatinib, relapse suggested kinase‐independent escape; treatment was redirected towards immune‐mediated clearance with one cycle of continuous blinatumomab plus dasatinib, achieving deep molecular remission. Allogeneic transplantation was deferred because of advanced age, prior cardiac dysfunction and cumulative infectious burden, and outpatient maintenance was initiated with monthly 24‐h blinatumomab infusions (35 μg) plus dasatinib (100 mg daily) and subcutaneous interferon alfa‐2b (3 MIU thrice weekly). Morphologic remission was sustained for over 3.5 years with no unplanned hospitalizations and no grade ≥3 neurotoxicity. *BCR::ABL1* transcripts remained undetectable for nearly 3 years before a transient low‐level fluctuation (0.0024% IS) appeared and stabilized without treatment escalation. Peripheral monitoring showed persistent CD8^+^ predominance and sustained B‐cell depletion (<20 cells/μL), indicating ongoing target engagement. Public transcriptomic data supported that intermittent bispecific exposure preserves progenitor/memory T‐cell programmes while limiting exhaustion.

**Conclusion:**

Intermittent reduced‐intensity blinatumomab maintenance guided by serial MRD monitoring was feasible and associated with durable molecular control in a transplant‐ineligible patient with high‐risk Ph^+^ ALL. This strategy merits prospective evaluation.

## Introduction

B‐lymphoblastic leukaemia/lymphoma with *BCR::ABL1* fusion, classically referred to as Philadelphia chromosome‐positive acute lymphoblastic leukaemia (Ph^+^ ALL), is a high‐risk adult acute leukaemia subtype associated with poor overall survival and rapid chemo‐resistance.^1^ Although multi‐generation tyrosine kinase inhibitors (TKIs) have improved frontline outcomes, patients with adverse genetic lesions—among them large *IKZF1* deletions that ablate Ikaros function—remain at high risk for relapse.^2^, ^3^ Allogeneic haematopoietic stem cell transplantation (allo‐HSCT) is the standard consolidation strategy to ensure durable molecular clearance.^1^ However, advanced age, medical comorbidities or poor performance status render a significant proportion of adult patients ineligible for transplantation.^4^

Maintaining long‐term measurable residual disease (MRD) negativity in transplant‐ineligible patients is challenging.^5^ While blinatumomab induces deep molecular responses, standard protocols require multiple cycles of 28‐day continuous intravenous infusion.^6^ This continuous exposure imposes a substantial logistical burden, carries a cumulative neurotoxicity risk and may drive T‐cell exhaustion.^4^, ^7^ Implementing treatment‐free intervals through an intermittent dosing schedule could mitigate these limitations.^8^ We report a 3.5‐year follow‐up of a 64‐year‐old transplant‐ineligible patient with *IKZF1*‐deleted Ph^+^ ALL who relapsed during dasatinib therapy with an acquired E255K/V mutation, and who achieved durable molecular control using an individualised, intermittent blinatumomab maintenance regimen.

## Methods

### MRD quantification (*BCR::ABL1*)

*BCR::ABL1* transcripts (p210) were monitored by real‐time quantitative reverse‐transcription PCR on bone marrow aspirates in the institutional clinical laboratory. Results were reported as the *BCR::ABL1/ABL1* ratio (%) and converted to the International Scale (IS) using a laboratory‐specific conversion factor calibrated against an external reference laboratory. The analytical sensitivity (limit of detection, LOD) of the assay was 0.001% IS; ‘undetectable’ was defined as a value below this assay‐specific LOD. Serial measurements were obtained under the same reporting format and interpreted in conjunction with concurrent bone marrow morphology and flow cytometric MRD.

### Flow cytometric MRD

Measurable residual disease was also assessed on bone marrow aspirates by multiparameter flow cytometry using an institutional B‐ALL MRD panel (8–10 colours). MRD negativity was defined as fewer than 0.01% abnormal B lymphoblasts among nucleated cells, consistent with laboratory reporting standards. Interpretation incorporated contemporaneous morphology and molecular testing.

### ABL1 kinase domain mutation testing

ABL1 kinase domain mutation analysis was performed on bone marrow samples using a validated sequencing assay in the institutional molecular diagnostics laboratory. Assay sensitivity and variant reporting followed laboratory validation standards.

### Bioinformatic analysis of public transcriptomic data

Publicly available transcriptomic data from GSE196463 were used to compare T‐cell gene expression profiles between continuous and intermittent bispecific antibody exposure. Raw count data were processed and normalised as described previously.^8^ Differential expression was assessed using a moderated *t*‐test with false discovery rate (FDR) correction; genes with FDR < 0.05 and |log_2_ fold change| > 0.5 were considered significant. T‐cell subtype abundance was estimated by computational deconvolution based on published signatures. All analyses were performed in R (version 4.5.2).

## Results

### Case presentation

A 64‐year‐old man presented in October 2020 with fatigue, petechiae, gingival bleeding and low‐grade fever. Laboratory evaluation showed leukocytosis (39.08 × 10^9^/L), anaemia (haemoglobin 9.2 g/dL), thrombocytopenia (12 × 10^9^/L) and 55% peripheral blood blasts. Bone marrow examination revealed 69.2% lymphoblasts, with immunophenotyping positive for CD19, CD22, cytoplasmic CD79a, CD34, HLA‐DR, and partially positive for CD10, alongside aberrant CD13 and CD33 expression. Molecular testing identified a *BCR::ABL1* p210 fusion transcript and a heterozygous *IKZF1* deletion (exons 1–8). Cytogenetic analysis confirmed a 46,XY,t(9;22)(q34;q11) translocation, establishing a diagnosis of high‐risk Ph^+^ ALL.

Frontline induction with dasatinib, vindesine and prednisone was initiated in October 2020, achieving morphologic complete remission by November 2020. However, molecular clearance was suboptimal, with a persistent marrow MRD of 0.2% by flow cytometry and a *BCR::ABL1* transcript level of 4.7452% on the IS. During consolidation through July 2021, the patient experienced recurrent infections and transient acute heart failure, while *BCR::ABL1* transcript levels rose to 10.0628% IS. Haematologic relapse occurred in August 2021, characterised by 14% bone marrow lymphoblasts, a *BCR::ABL1* level of 73.2196% IS and an acquired ABL1 domain E255K/V mutation (Table 1).

**Table 1:** Milestone‐based disease trajectory, molecular monitoring and therapeutic interventions

Date	Disease status	Laboratory and molecular monitoring	Treatment
Diagnosis and induction			
Oct 2020	Newly diagnosed	BM blasts 69.2%; MRD‐positive; *BCR::ABL1* p210 positive; *IKZF1* del ex1–8; 46,XY,t(9;22)	D‐VP induction
First remission and consolidation			
Nov 2020	CR1	BM‐negative; MRD 0.2%; IS 4.7452%	D‐VP consolidation
Dec 2020–Jul 2021	CR1 with persistent MRD	MRD‐positive throughout; IS nadir 0.0421% (Apr), rising to 10.0628% (Jul)	D‐VP × 5; dasatinib + IFN‐α2b
Complications	–	Invasive aspergillosis, CMV pneumonia, *Clostridioides difficile–*associated colitis, transient heart failure; ≈15 hospital admissions	–
First relapse and salvage			
Aug 2021	**1st relapse**	BM blasts 14.0%; MRD 19.0% (FCM); IS 73.2196%; E255K/V mutation	D‐VP re‐induction; dasatinib intensified
Sep 2021	CR2 (MRD‐positive)	BM‐negative; MRD 2.1%; IS 84.3640%	Dasatinib
Oct 2021	CMR	BM‐negative; MRD‐negative; IS 0.0000%	Blinatumomab 9 → 28 μg/d (d1–21) + dasatinib
Allo‐HSCT	Deferred	–	Age, comorbidities, prior infections
Reduced‐intensity maintenance			
Nov 2021–Apr 2025	Sustained remission with late‐stage low‐level molecular fluctuation	BM and flow MRD‐negative throughout; IS negative until Sep 2024, followed by a transient low‐level fluctuation (0.0024% IS) late in the follow‐up period	Blinatumomab 35 μg q4wk (24‐h infusion, day‐hospital) + dasatinib + IFN‐α2b
Clinical burden	–	No unplanned hospitalizations; no CRS; no grade ≥ 3 neurotoxicity	–

*Note:* The bold font is used to highlight key molecular events and clinically important time points for the reader, specifically the emergence of the ABL1 E255K/V mutation and the late‐stage transient low‐level BCR::ABL1 fluctuation (0.0024% IS).

Allo‐HSCT, allogeneic haematopoietic stem cell transplantation; BM, bone marrow; CMR, complete molecular response; CR, complete remission; CRS, cytokine release syndrome; FCM, flow cytometry; IFN, interferon; IS, International Scale; MRD, measurable residual disease; VP, vindesine and prednisone.

The E255K/V substitution is a P‐loop mutation that confers resistance to imatinib and nilotinib, whereas dasatinib and ponatinib retain activity against this mutant.^9^, ^10^ Notably, the patient relapsed while receiving dasatinib—a drug to which E255K/V‐bearing clones are expected to remain sensitive. This clinical course suggested that kinase‐independent mechanisms contributed to the relapse and that simply substituting one TKI for another was unlikely to offer a decisive advantage. After discussion with the patient, treatment was redirected towards immune‐mediated clearance. The patient was transitioned to an immune‐targeted salvage regimen combining one cycle of continuous blinatumomab (9 μg/d step‐up to 28 μg/d, Days 1–21) with dasatinib, achieving morphologic second complete remission (CR2), flow cytometric MRD negativity and undetectable *BCR::ABL1* transcripts by October 2021.

In November 2021, an intermittent maintenance protocol was initiated, consisting of brief, 24‐h monitored infusions of blinatumomab (35 μg administered approximately monthly in an outpatient day‐hospital setting). Dasatinib was continued at its standard dose (100 mg daily) for its immunomodulatory properties, rather than as the primary antileukaemic agent, alongside subcutaneous recombinant interferon alfa‐2b (3 MIU thrice weekly; Table 1). The 35 μg dose corresponded to the single‐vial formulation of blinatumomab, allowing the full contents of one vial to be administered at each visit and avoiding wastage of this costly agent. The dosing interval of approximately 1 month was chosen to provide periodic tumour‐directed immune activation while allowing T‐cell recovery, based on the premise that continuous bispecific exposure drives terminal T‐cell exhaustion.^8^ Morphologic remission was maintained for over 3.5 years in an entirely outpatient setting, with no unplanned hospitalizations and no grade ≥3 neurotoxicity. Quantitative PCR detected a late‐stage low‐level *BCR::ABL1* transcript fluctuation (0.0024% IS), which remained stable under outpatient surveillance.

These clinical and molecular dynamics are chronologically detailed in Table 1. To contextualise this individualised schedule, the regimen was compared with published blinatumomab protocols across frontline, MRD‐positive and relapsed/refractory settings (Supplementary table 1).^2^, ^3^, ^6^, ^11^, ^12^ While standard published regimens rely on continuous multi‐week intravenous infusions requiring ambulatory pumps and prolonged or repeated hospitalisations, this protocol utilised discrete 24‐h infusions at monthly intervals. This approach reduced the cumulative treatment and financial burden while maintaining molecular disease control.

### Longitudinal molecular and immunologic dynamics

**Figure 1:**
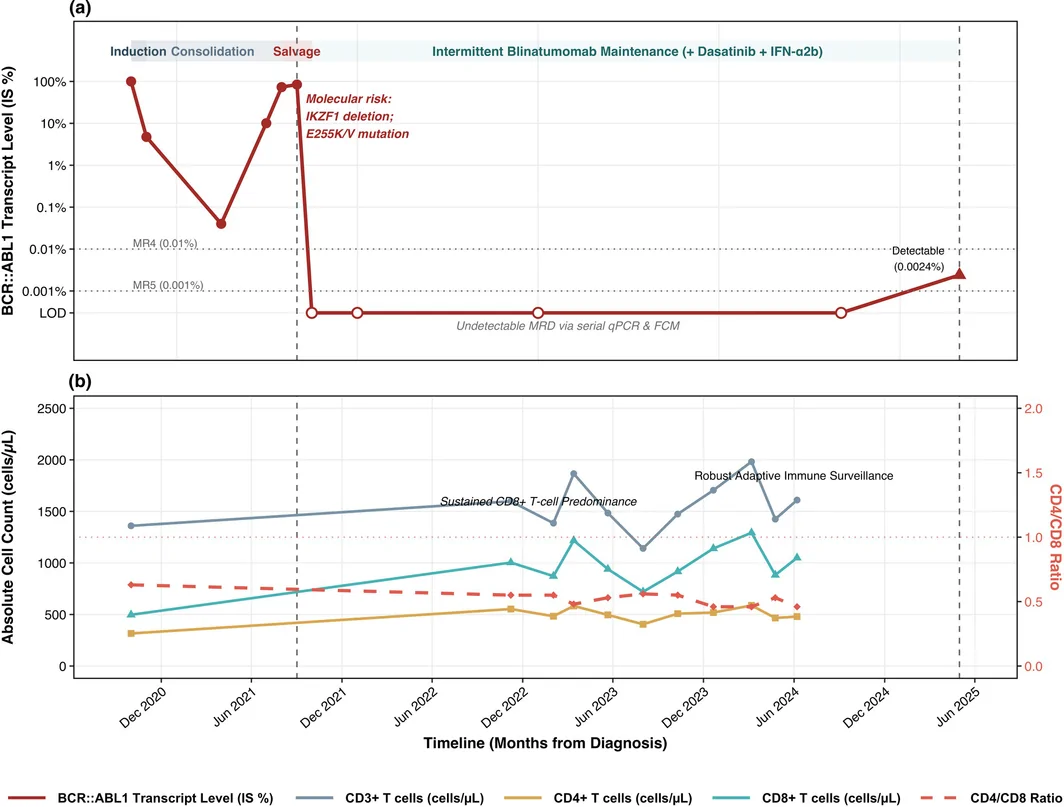
Longitudinal disease monitoring and descriptive immune profile during post‐remission follow‐up. **(a)** Longitudinal *BCR::ABL1* transcript levels (International Scale, %) across the disease course, including induction therapy, relapse, salvage treatment and post‐remission management. Undetectable values indicate transcript levels below the assay‐specific limit of detection. **(b)** Descriptive peripheral blood T‐cell subset profile during post‐remission follow‐up. Absolute counts of CD3^+^ T cells, CD4^+^ T cells, CD8^+^ T cells and the CD4^+^/CD8^+^ ratio are shown at selected time points during maintenance.

Serial real‐time PCR tracking showed that *BCR::ABL1* transcripts remained below the limit of detection for nearly 3 years following salvage therapy (Figure 1a). A low‐level transcript fluctuation was detected late in the follow‐up period (Figure 1a). Without cytotoxic re‐induction or adjustment to the monthly 24‐h blinatumomab schedule, the transcript level remained stable through the May 2025 data cut‐off (Figure 1a).

Peripheral lymphocyte tracking during this period showed a stable CD8^+^‐predominant T‐cell profile (Figure 1b). Absolute CD8^+^ T‐cell counts remained elevated during remission, with minor fluctuations matching the late‐stage molecular data before plateauing. The peripheral CD4^+^/CD8^+^ ratio remained inverted (< 1.0; Figure 1b). Notably, the peripheral B‐cell compartment was continuously suppressed (< 20 cells/μL), reflecting sustained *in vivo* target engagement by the bispecific antibody despite the prolonged treatment‐free intervals.

### Bioinformatic evaluation of exposure cadence

**Figure 2:**
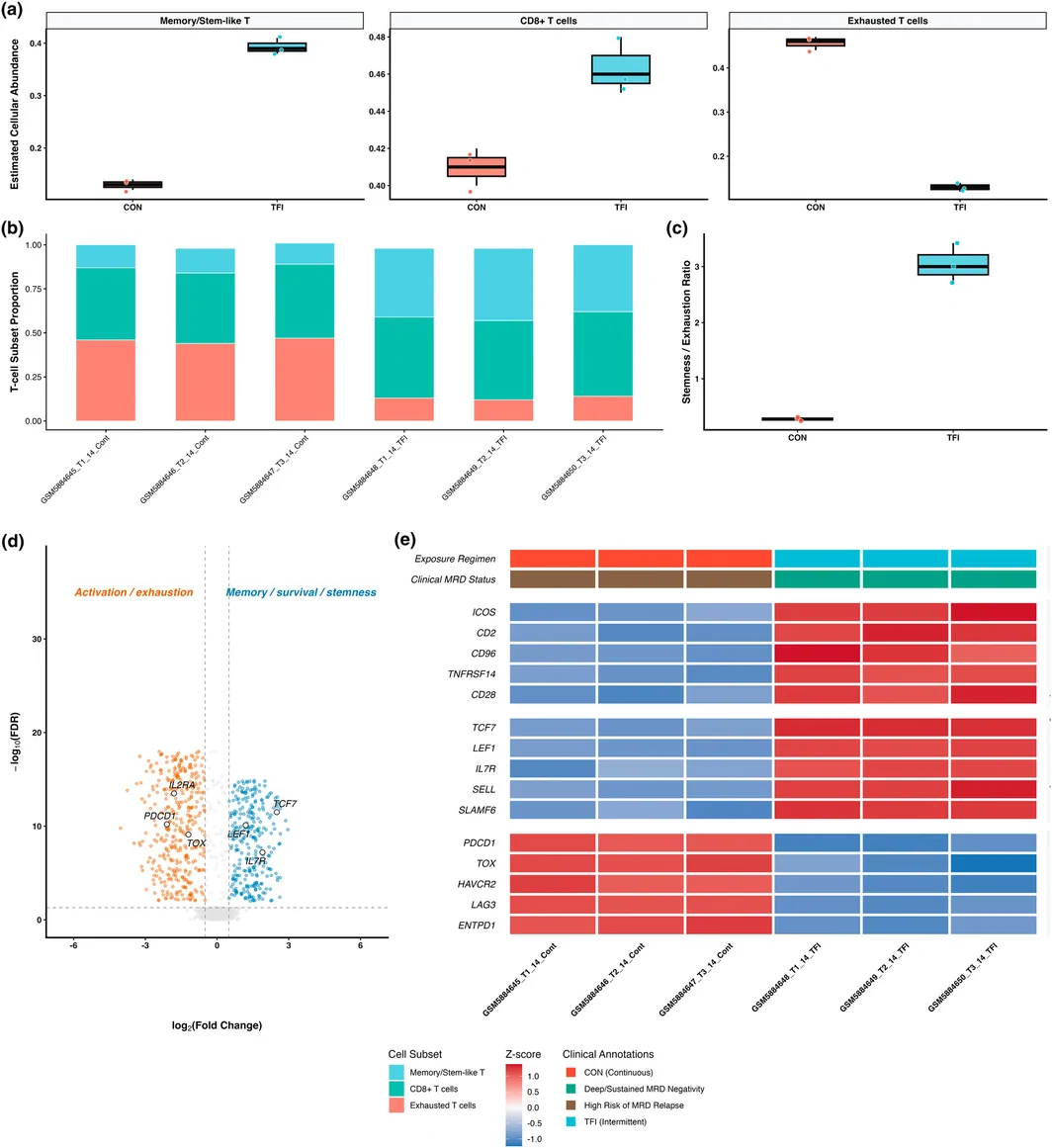
T‐cell ecosystem remodelling and functional fate dynamics during MRD clearance. **(a)** Estimated abundance of functional T‐cell states under continuous (CON) versus intermittent (TFI, treatment‐free intervals) blinatumomab exposure. Data are represented as boxplots overlaid with paired lines connecting individual donors (*n* = 3). Statistical significance evaluated via an unadjusted paired two‐tailed Student's *t*‐test (*P* < 0.05, *P* < 0.01, ns, not significant). **(b)** Stacked bar profiles illustrating the shift in relative proportions of T‐cell sub‐populations across donor‐matched pairs (*n* = 3 donors) at Day 14 post‐treatment. **(c)** Shift in the stemness‐to‐exhaustion ratio between matched exposure conditions (paired two‐tailed Student's *t*‐test; exact *P*‐values indicated within the panel). **(d)** Differential gene expression between treatment regimens. Labelled data points highlight key differentially expressed genes (DEGs) associated with memory/stemness (blue) or activation/exhaustion (orange) programs; dashed lines indicate significance thresholds (FDR < 0.05, |log_2_ FC| > 0.5). **(e)** Integrated expression matrix cross‐referenced with clinical tracks. Upper horizontal bars correspond to the discrete clinical legend indicating exposure regimens and minimal residual disease (MRD) status phenotypes. Lower matrix displays row‐scaled transcriptomic *Z*‐scores partitioned into distinct functional modules (CD8^+^ Clearance Capacity, Progenitor/Memory Stemness, and Terminal Exhaustion/Resistance).

To evaluate the microenvironmental effects of divergent blinatumomab exposure kinetics, raw transcriptomic data from donor‐matched bone marrow samples (GSE196463) were analysed.^8^ Computational deconvolution of T‐cell subsets showed distinct immunophenotypic profiles dictated by the drug exposure cadence. Continuous bispecific antibody exposure increased the transcriptomic abundance scores of effector CD8^+^ signatures but expanded exhaustion pathways. Conversely, the intermittent regimen preserved the progenitor/memory lineage compartment, increasing the calculated stemness‐to‐exhaustion ratio index (Figure 2a–c).

Global differential gene expression profiling showed distinct transcriptomic profiles based on the drug delivery schedule (Figure 2d).^8^ Continuous infusion upregulated terminal activation‐exhaustion regulators, including *IL2RA*, *PDCD1* and *TOX*. The intermittent schedule maintained expression of genes associated with memory longevity and stemness, such as *TCF7*, *LEF1* and *IL7R* (FDR < 0.05, |log_2_ FC| > 0.5).^8^ Cross‐referencing these functional modules with clinical tracks demonstrated that the intermittent schedule was associated with the upregulation of both the CD8^+^ Clearance Capacity and Progenitor/Memory Stemness functional blocks (Figure 2e).^8^ Continuous blinatumomab exposure correlated primarily with Terminal Exhaustion/Resistance modules, indicating that insertional treatment‐free windows preserve the transcriptional fitness required for long‐term minimal residual disease control.^6^, ^8^

## Discussion

Sustaining deep molecular remission in transplant‐ineligible patients with high‐risk Ph^+^ ALL is clinically challenging, particularly when an *IKZF1* deletion coexists with an acquired ABL1 kinase domain mutation that emerges during active therapy.^1^, ^13^, ^14^ Heterozygous deletion of *IKZF1* alters the IKAROS zinc‐finger transcription factor, promoting an immature, adhesive phenotype in B‐progenitor blasts that facilitates microenvironmental nesting within the bone marrow niche, leading to clonal escape under standard therapy.^14^ In this patient, standard frontline dasatinib consolidation failed to achieve durable molecular clearance, with relapse accompanied by emergence of the ABL1 E255K/V substitution.^2^, ^14^

The choice of therapy after E255K/V was documented deserves comment. This P‐loop mutation confers resistance to imatinib and nilotinib but remains sensitive to dasatinib and ponatinib.^9^, ^10^ The patient nonetheless relapsed while receiving dasatinib—a drug to which the mutant clone should, in principle, have remained sensitive. This dissociation between predicted *in vitro* sensitivity and the observed clinical course argues that kinase‐independent escape mechanisms were likely at play, and that a simple switch between TKIs was unlikely to restore durable control. Ponatinib, although active against E255K/V, was further constrained by its well‐documented risk of arterial occlusive events in a 64‐year‐old patient with prior transient heart failure. After discussion with the patient, the strategy was therefore deliberately shifted from kinase dependence towards immune‐mediated surveillance; dasatinib was continued for its immunomodulatory properties rather than as a primary antileukaemic agent. The durable molecular control achieved with the subsequent blinatumomab‐based protocol supports this approach. When oncogene‐targeted kinase inhibition is compromised, disease control can be shifted towards an immune‐mediated surveillance framework.^2^, ^3^, ^7^ The response to this maintenance platform likely stems from multi‐agent dynamics between the three components.^3^, ^11^ While standard protocols utilise multi‐week continuous blinatumomab infusions that can cause cumulative systemic toxicities and chronic T‐cell overstimulation,^2^, ^3^, ^6^, ^11^, ^12^ the intermittent 24‐h infusion schedule provides periodic immune engagement while minimising receptor overstimulation.^8^

This delivery window was supported by the co‐administration of interferon alfa‐2b and dasatinib.^3^, ^11^ Recombinant interferon alfa‐2b can expand and sustain memory‐like CD8^+^ T‐cell clones, compensating for the absence of continuous bispecific target pressure. Meanwhile, dasatinib retains the capacity to modulate the immune microenvironment by suppressing regulatory T cells and enhancing effector trafficking^2^, ^3^; these immunomodulatory effects operate independently of direct kinase inhibition and are therefore not expected to be nullified by the E255K/V substitution. The choice of the 35 μg blinatumomab dose was largely practical: It corresponds to the single‐vial strength of the product, which permitted full administration without wastage and simplified outpatient delivery. This pragmatic basis should not obscure the biologic rationale for the schedule itself, which was designed to exploit the short plasma half‐life of blinatumomab and create deliberate treatment‐free intervals. At the same time, the optimal dose intensity and duration of bispecific T‐cell engagers across different disease burdens remain incompletely defined, and it is neither practical nor realistic to conduct separate randomised dose‐finding trials for every de‐escalated maintenance regimen. In this regard, the persistent B‐cell depletion (< 20 cells/μL) observed throughout the follow‐up period provides an indirect pharmacodynamic readout of sustained target engagement, thereby offering a functional anchor for the selected dose. More broadly, when a sensitive, disease‐specific MRD marker such as *BCR::ABL1* qPCR is available, it can serve as a guide for tailoring the intensity and duration of immune‐based maintenance; this MRD‐directed approach is increasingly being explored in immunotherapy for ALL. In addition, intermittent dosing, by reducing cumulative drug exposure, may help limit the long‐term immunosuppressive burden and the associated risk of opportunistic infections—an important consideration for patients receiving prolonged maintenance. This multi‐agent mechanism is consistent with transcriptomic data from Philipp et al. (GSE196463), which demonstrated that continuous bispecific overstimulation upregulates terminal resistance pathways, whereas treatment‐free intervals preserve the transcriptional fitness of progenitor and memory stemness circuits.^8^

The longitudinal clinical tracking captured a late‐stage transient molecular transcript fluctuation (0.0024% IS), which stabilised under close observation. This event reflects the micro‐evolutionary volatility of *IKZF1*‐deleted clones, but indicates that the host T‐cell compartment retained the capacity to contain low‐level disease fluctuations without immediate cytotoxic chemotherapy.^7^, ^8^ At diagnosis, the leukaemic clone was defined by a 46,XY,t(9;22)(q34;q11) translocation and a heterozygous *IKZF1* deletion involving exons 1–8. Sequential conventional karyotyping and dedicated *IKZF1* deletion testing were not repeated after deep molecular remission was achieved; the monitoring program for this patient relied primarily on serial *BCR::ABL1* qPCR and flow cytometric MRD. The sustained *BCR::ABL1* negativity and flow MRD negativity over 3.5 years nonetheless argue against persistence of the original leukaemic clone.

This study has several limitations. It describes a single patient receiving a tailored, non‐standardised regimen, so the individual quantitative contributions of blinatumomab, dasatinib and interferon cannot be statistically isolated. Additionally, high‐sensitivity MRD assays by next‐generation sequencing (NGS; e.g. clonoSEQ) were not available at our institution during the study period; MRD monitoring relied on flow cytometry and *BCR::ABL1* qPCR, the latter being the standard high‐sensitivity method for Ph^+^ ALL with an LOD of 0.001% IS. The immunophenotypic tracking was descriptive, and the transcriptomic data were obtained from an external trial cohort rather than patient‐specific multi‐omic profiles. Furthermore, although cytogenetic and *IKZF1*‐specific testing was performed at diagnosis, sequential reassessment was not part of the routine MRD program at our centre, a limitation that should be addressed in future prospective studies. Despite these limitations, this case demonstrates that shifting bispecific antibody delivery from continuous to intermittent kinetics minimises persistent receptor overstimulation, optimising T‐cell fitness in selected high‐risk, transplant‐ineligible patients.^6^, ^8^

## Conclusion

An intermittent, reduced‐intensity blinatumomab maintenance strategy integrated within a multi‐agent immune‐targeted backbone was clinically feasible and well‐tolerated in a transplant‐ineligible patient with high‐risk Ph^+^ ALL harbouring an *IKZF1* deletion and an ABL1 kinase domain mutation. The prevention of haematologic relapse, despite adverse genetic biology and a transient transcript fluctuation, suggests that optimising drug delivery kinetics to maintain host T‐cell fitness is a viable therapeutic objective. Prospective, multi‐centre trials integrating real‐time MRD kinetics and high‐dimensional immune profiling are required to validate this de‐escalated maintenance approach.

## Author contributions

**Xiaoqian Duan:** Writing – original draft; conceptualization; data curation; methodology. **Jinjun Yang:** Conceptualization; methodology; writing – review and editing; data curation. **Yufei Yang:** Software. **Shuwen Guo:** Software. **Yu Wu:** Conceptualization; funding acquisition; writing – review and editing; supervision.

## Conflict of interest

The authors declare no conflict of interest.

## Funding information

This work was supported by the National Natural Science Foundation of China (No. 82370171) and the 2025 Clinical Research Youth Fund of West China Hospital, Sichuan University (2025HXFH025).

## Ethics statement

This study was conducted in compliance with the ethical principles of the Declaration of Helsinki and was approved by the Institutional Review Board of West China Hospital, Sichuan University.

## Patient consent statement

The patient provided written informed consent for the collection and use of their clinical data.

## Supporting information


Supplementary table 1

## Data Availability

The datasets generated or analysed in this study are available from the corresponding author upon reasonable request.

## References

[1] Gökbuget N , Boissel N , Chiaretti S *et al*. Management of ALL in adults: 2024 ELN recommendations from a European expert panel. Blood 2024; 143: 1903–1930. 10.1182/blood.2023023568 38306595

[2] Foà R , Bassan R , Vitale A *et al*. Dasatinib‐blinatumomab for Ph‐positive acute lymphoblastic leukemia in adults. N Engl J Med 2020; 383: 1613–1623. 10.1056/NEJMoa2016272 33085860

[3] Jabbour E , Short NJ , Jain N *et al*. Ponatinib and blinatumomab for Philadelphia chromosome‐positive acute lymphoblastic leukaemia: A US, single‐centre, single‐arm, phase 2 trial. Lancet Haematol 2023; 10: e24–e34. 10.1016/S2352-3026(22)00319-2 36402146

[4] Russell‐Smith A , Murphy L , Nguyen A *et al*. Real‐world use of inotuzumab ozogamicin is associated with lower health care costs than blinatumomab in patients with acute lymphoblastic leukemia in the first relapsed/refractory setting. J Comp Eff Res 2024; 13: e230142. 10.57264/cer-2023-0142 PMC10842295 38099517

[5] Kantarjian H , Stein A , Gökbuget N *et al*. Blinatumomab versus chemotherapy for advanced acute lymphoblastic leukemia. N Engl J Med 2017; 376: 836–847. 10.1056/NEJMoa1609783 PMC5881572 28249141

[6] Gökbuget N , Dombret H , Bonifacio M *et al*. Blinatumomab for minimal residual disease in adults with B‐cell precursor acute lymphoblastic leukemia. Blood 2018; 131: 1522–1531. 10.1182/blood-2017-08-798322 PMC6027091 29358182

[7] Chiaretti S , Foà R . How I treat adult Ph^+^ ALL. Blood 2025; 145: 11–19. 10.1182/blood.2023023152 39172753

[8] Philipp N , Kazerani M , Nicholls A *et al*. T‐cell exhaustion induced by continuous bispecific molecule exposure is ameliorated by treatment‐free intervals. Blood 2022; 140: 1104–1118. 10.1182/blood.2022015956 PMC10652962 35878001

[9] Soverini S , Hochhaus A , Nicolini FE *et al*. BCR‐ABL kinase domain mutation analysis in chronic myeloid leukemia patients treated with tyrosine kinase inhibitors: Recommendations from an expert panel on behalf of European LeukemiaNet. Blood 2011; 118: 1208–1215. 10.1182/blood-2010-12-326405 21562040

[10] Hochhaus A , Baccarani M , Silver RT *et al*. European LeukemiaNet 2020 recommendations for treating chronic myeloid leukemia. Leukemia 2020; 34: 966–984. 10.1038/s41375-020-0776-2 PMC7214240 32127639

[11] Foà R , Bassan R , Elia L *et al*. Long‐term results of the dasatinib‐blinatumomab protocol for adult Philadelphia‐positive ALL. J Clin Oncol 2024; 42: 881–885. 10.1200/JCO.23.01075 PMC10927329 38127722

[12] Martinelli G , Boissel N , Chevallier P *et al*. Complete hematologic and molecular response in adult patients with relapsed/refractory Philadelphia chromosome‐positive B‐precursor acute lymphoblastic leukemia following treatment with blinatumomab: Results from a phase II, single‐arm, multicenter study. J Clin Oncol 2017; 35: 1795–1802. 10.1200/JCO.2016.69.3531 28355115

[13] Short NJ , Jabbour E , Sasaki K *et al*. Impact of complete molecular response on survival in patients with Philadelphia chromosome‐positive acute lymphoblastic leukemia. Blood 2016; 128: 504–507. 10.1182/blood-2016-03-707562 PMC4965905 27235138

[14] Martinelli G , Iacobucci I , Storlazzi CT *et al*. *IKZF1* (Ikaros) deletions in BCR‐ABL1‐positive acute lymphoblastic leukemia are associated with short disease‐free survival and high rate of cumulative incidence of relapse: A GIMEMA AL WP report. J Clin Oncol 2009; 27: 5202–5207. 10.1200/JCO.2008.21.6408 19770381

