# Supplementary material for: Prolonged molecular control during intermittent reduced‐intensity blinatumomab maintenance in a transplant‐ineligible patient with Philadelphia chromosome‐positive acute lymphoblastic leukaemia and IKZF1 deletion
Source: Clin Transl Immunology. 2026 Sep 21;15(9):e70121. doi: 10.1002/cti2.70121 (PMC13594394; doi:10.1002/cti2.70121)
Supplement: Supplementary file 1 [file CTI2-15-e70121-s001.docx]

**Supplementary Material**

**Supplementary Table 1. Clinical context of standard blinatumomab schedules and the individualized reduced-intensity strategy used in the present case**

| **Reference** | **Clinical setting** | **Treatment context** | **Exposure pattern** | **Delivery and safety** | **Relevance to present case** |
| --- | --- | --- | --- | --- | --- |
| Foà et al., NEJM 2020 (D-ALBA) [2] | Newly diagnosed adult Ph^+^ ALL | Post-induction consolidation (dasatinib + glucocorticoids) | Continuous IV infusion (28 μg/day; 2–5 cycles) | Continuous pump-based delivery; inpatient or ambulatory; grade ≥3 adverse events | Establishes standard continuous multi-cycle framework; no data on reduced-intensity maintenance |
| Foà et al., J Clin Oncol 2024 [11] | Long-term follow-up of D-ALBA cohort | Dasatinib–blinatumomab platform | Continuous IV infusion (multi-cycle regimen) | Standard infusion delivery; manageable long-term toxicity; no de-escalation strategy | Confirms durability; no data on intermittent maintenance |
| Jabbour et al., Lancet Haematol 2023 [3] | Frontline adult Ph^+^ ALL | Chemotherapy-free induction/consolidation (ponatinib-based) | Continuous IV infusion (multi-cycle regimen) | Continuous infusion management required | High-efficacy comparator; relies on continuous rather than intermittent exposure |
| Gökbuget et al., Blood 2018 (BLAST) [6] | MRD-positive B-cell precursor ALL (CR) | MRD-directed post-remission therapy | Continuous IV infusion (15 μg/m²/day; up to 4 cycles; 4 weeks on/2 weeks off) | Pump-dependent delivery; CRS and grade 3/4 neurotoxicity reported | Supports MRD-directed therapy; no prolonged maintenance data |
| Martinelli et al., J Clin Oncol 2017 (ALCANTARA) [12] | Relapsed/refractory Ph^+^ ALL (post-TKI failure) | Single-agent salvage | Continuous IV infusion (9 μg/day week 1 → 28 μg/day; up to 5 cycles) | Pump-based delivery; grade 1–2 CRS; grade 3 neurotoxicity | Defines salvage setting; no data on de-escalated maintenance |
| Chiaretti & Foà, Blood 2025 [7] | Adult Ph^+^ ALL (expert review) | TKI- and blinatumomab-based strategies | Continuous IV infusion (standard 2–5 cycles) | Overview of standard delivery and safety constraints | Highlights lack of evidence for long-term maintenance strategies |
| Present case | Transplant-ineligible high-risk Ph^+^ ALL (*IKZF1* deletion, ABL1 E255K/V) | Salvage followed by individualized post-remission maintenance | Intermittent IV infusion (35 μg/dose; approximately monthly; 24-h monitored) | Day-hospital delivery; no ambulatory pump; no CRS; no grade ≥3 neurotoxicity | Illustrates an individualized intermittent strategy; provides a reduced-intensity alternative to continuous exposure |

Abbreviations: ALL, acute lymphoblastic leukemia; CR, complete remission; CRS, cytokine release syndrome; IV, intravenous; MRD, measurable residual disease; Ph^+^, Philadelphia chromosome-positive; TKI, tyrosine kinase inhibitor.
